# “They can rest at home”: an observational study of patients’ quality of sleep in an Australian hospital

**DOI:** 10.1186/s12913-018-3201-z

**Published:** 2018-07-05

**Authors:** Lori J. Delaney, Marian J. Currie, Hsin-Chia Carol Huang, Violeta Lopez, Frank Van Haren

**Affiliations:** 1 Faculty of Nursing, University of Canberra, Canberra, Australia; 2 College of Health and Medicine, Australian National University, Canberra, Australia; 3 Respiratory and Sleep Medicine, Canberra Hospital, Garran, Australia; 4 Alice Lee Centre for Nursing Studies, Yong Loo Lin School of Medicine, Singapore, Singapore; 5 Intensive Care Unit, Canberra Hospital, Garran, Australia; 6 Faculty of Health, Discipline of Nursing and Midwifery, University of Canberra, Canberra, ACT 2601 Australia

**Keywords:** Clinical care, Environmental stressor, Hospital, Medical, Noise, Nursing, Sleep, Sleep disturbance

## Abstract

**Background:**

Poor sleep is known to adversely affect hospital patients’ recovery and rehabilitation. The aim of the study was to investigate the perceived duration and quality of patient sleep and identify any environmental factors associated with patient-reported poor sleep in hospital.

**Method:**

A cross-sectional study was conducted involving 15 clinical units within a 672-bed tertiary-referral hospital in Australia. Semi-structured interviews to determine perceptions of sleep quantity and quality and factors that disturb nocturnal sleep were conducted with patients and nursing staff. Environmental noise, light and temperature were monitored overnight, with concurrent logging of noise sources by observers.

**Results:**

Patients reported a mean reduction in hospital sleep duration, compared to home, of 1.8 h (5.3 vs. 7.1 h; *p* < 0.001). The proportions of patients reporting their sleep quality to be poor/very poor, fair and of good quality were 41.6, 34.2 and 24.2% respectively. Patients reported poorer sleep quality than nurses (*p* < 0.05). Patients, nurses and observers all reported the main factors associated with poor sleep as clinical care interventions (34.3%) and environmental noise (32.1%). Noise levels in all 15 clinical areas exceeded WHO recommended levels of < 30 dB [A] by 36.7 to 82.6%, with peak noise levels of 51.3 to 103.3 dB (A).

**Conclusion:**

Hospital in-patients are exposed to factors which reduce the duration and quality of their sleep. These extrinsic factors are potentially modifiable through behaviour change and reconfiguration of the clinical environment. The findings from this study provided the foundation for a quality improvement project currently underway to improve patients’ sleep.

## Background

There is evidence that poor sleep in hospital jeopardises patients’ recovery. The ability to sleep is important for somatic and cognitive processing, and physiological recovery. Sleep disturbance has deleterious effects on immunological function, [1, 2] the hypothalamic-pituitary-adrenocortical and somatotropic systems, [3–7] and can decrease responsiveness to hypoxic states and inspiratory muscle strength [8, 9]. Neurobehavioural alterations such as confusion, delirium and a decline in working memory have also been attributed to sleep disturbance [10–13]. These effects have been reported to persist even after recovery sleep has been obtained, [11] negating the idea that the effects of sleep disturbance can be overcome once patients are discharged from hospital or high acuity areas.

Sleep disturbance in the clinical environment has been attributed to several extrinsic factors such as ambient noise, exposure to artificial lighting and clinical interactions [13–17]. Noise has been the most studied sleep disturbing factor with reported noise levels frequently exceeding the World Health Organization (WHO) recommended nocturnal noise levels of less than 30 dB (A) in the clinical environment [18]. Studies report ambient noise levels ranging from 50 to 60 dB(A) in intensive care, [13, 15, 19] and 40 to 55 dB(A) in the general ward environment [20].

The clinical environment may also influence the circadian rhythm of sleep as artificial lighting can suppress melatonin production. Melatonin plays an important regulatory role in sleep–wake patterns, and exposure to artificial light during the sleep phase can adversely affect patients’ perceptions of the quality of their sleep [21–23]. Environmental temperature has also been identified as a factor that can induce misalignment of sleep–wake cycles, which can affect the restorative sleep phases of slow wave and rapid eye movement sleep [24–26].

Patients sleep in hospital has not been fully elucidated or explored in the Australian context. This study sought to determine the need for interventions to improve patients’ sleep in a large tertiary referral hospital in Australia, by determining sleep duration and identifying and quantifying the factors that adversely affect patient sleep in this setting.

## Methods

A prospective cross-sectional study was conducted over a six-month period (May to October 2013) in 15 clinical areas: six acute medical and six surgical wards, an intensive and high dependency care unit, and one aged care and one rehabilitation ward. These clinical areas were considered representative of services provided in this 672-bed tertiary referral teaching hospital in Australia. The study involved technical monitoring and concurrent direct observation of the nocturnal environment, patient interviews and nursing reports for each clinical unit for two consecutive nights between 22:00 and 07:00 h.

### Participants

Patients and nursing staff were recruited for this study. Non-probability convenience sampling was used to recruit patients to participate in a semi-structured interview to provide their individual perspective on sleep in the clinical setting. Patients admitted to any one of the 15 clinical wards on the two consecutive nights were eligible to participate in the interviews, unless they were intubated; were receiving end-of-life care; had a medical diagnosis of florid psychosis, dementia, confusion or expressive disorder; or the medical or nursing staff considered them clinically unsuitable.

Nursing staff working the night shift during the monitoring period were invited to complete a survey regarding their perceptions of the quality of their patients’ sleep and what they considered to be sleep-disturbing factors. Nursing staff were not required to provide direct patient care to the patients who participated in the semi-structured interview. Rather, nursing staff provided an overview of the clinical environment at the time of monitoring.

### Data collection

Data were collected using three methods: subjective data were obtained from nursing staff and patients, objective clinical data were derived from environmental monitoring and observational data were documented by the study research assistants. These data were concurrently obtained over two consecutive nights on each ward. The data collection tools were adapted, with approval, from documents designed by the Imperial College Healthcare (United Kingdom National Health Service) to more accurately reflect the Australian hospital’s clinical environment in terms of service delivery and design.

The researchers made no attempt to blind clinical staff or patients to the study purpose. Staff were informed that technical and observational information were being collected about the clinical environment in general, and not specifically about them or the patients. No alterations were made to the clinical environment, such as reminding staff to turn off lights or alerting them to alarms.

### Outcome measures

Four instruments were used to measure the study outcomes which had been developed by researchers at the Imperial College Healthcare (UK) based on existing sleep questionnaires and review of the literature: the Patient Sleep Interview Form [27], the Nurses’ Self-report Form [27], the Environmental Sleep Observation Form and environmental monitoring devices [27].

#### Patient sleep interview form

The patient interviews comprised open-ended questions concerning the patients’ perceptions of their sleep experience in the hospital. The interview questions addressed sleep quality; sleep hygiene behaviours at home and if and how their sleep differed in the hospital; and which factors they believed inhibited, prohibited or aided their sleep in the hospital. The patients were interviewed once the environmental monitoring of the clinical ward was completed, and their responses were recorded verbatim in writing by the research assistant conducting the interview.

#### Nurses’ self-report form

This questionnaire elicited nurses’ perceptions of their patients’ quality of sleep in the clinical ward, including the factors that they perceived inhibited the patients’ ability to sleep inclusive of the clinical environment. Nurses’ suggestions on how to improve patients’ sleep were also requested. Comments provided by clinical staff pertained generally to their clinical environment and were not matched to the patients who participated in the semi-structured interviews.

#### Environmental sleep observation form

The Environmental Sleep Observational Form was used to log the frequency of predetermined categorised noise sources which had the potential to inhibit or disturb patients’ sleep, such as conversations, alarms, telephone calls and patient buzzers. The documentation of observational data was completed by two research assistants (who were not registered nurses) during each night of clinical monitoring for one hour at four time points: 23:00, 02:00, 04:00 and 06:00 h. These timeframes were selected to reflect the spread of activity throughout the night in the clinical setting.

#### Environmental monitoring

Environmental monitoring devices were positioned in the clinical environment to monitor noise, luminance and temperature. A total of 12 noise, light and temperature monitors were positioned in a range of clinical areas in each ward, including the nursing station, corridors, and single and shared patient rooms in order to capture the activity in the clinical areas. The devices were positioned directly behind the patients’ beds, permitting the monitoring of stimuli from the patients’ perspective, while minimising their effect on the work of the clinical staff. In clinical areas, such as nursing stations and corridors, sound monitoring devices were mounted on the wall in the centre of the room. This occurred concurrently with the patient interviews and nursing surveys.

##### Noise and luminance monitoring

Noise and luminance levels were recording using Extech sound level meters (Model SDL600, frequency range 31.5 Hz to 8 KHz) and Extech light meters (Model SDL400), which both conformed to American National Standards Institute and International Electro-technical Commission Standards.

Noise levels were recorded in A-weighted decibels (dB), with a fast response time (125 ms) to permit the capture of peak noises (within a decibel Min-Max from 30 to 180 dB), and noises that occurred rapidly. The A-weighted filter was used because it attenuates the curve that describes loudness frequency for the human ear. Whilst luminance levels were recorded in Lux, with a recording Min-Max of 0 to 1999 Lux accuracy: +/− 4% + 2 disability glare threshold.

Noise and light meters were located within close proximity to each other and were programmed to log data at five-second epochs, and were calibrated, as per the manufacturer’s recommendations, before each recording. All logged data from the sound and luminance meters were saved to a secure digital 2 GB memory card in a spreadsheet format.

##### Temperature and humidity monitoring

Temperature and humidity were recorded at 30-s epochs using ThermoLoggers (Thermodata Pty Ltd) (accuracy +/− 1 °C and =/− 0.6% humidity, range temperature − 40 °C to + 85 °C), as temperature and humidity is less variable than the other environmental measures. These devices were calibrated prior to being positioned within the clinical environment as per manufacturers’ recommendation and were positioned within close proximity to the sound and luminance monitors. Data was then downloaded directly from the thermologgers into Excel (Microsoft 2010) spreadsheets.

### Data analysis

All data were collated, coded and entered into the Statistical Packages for Social Science (IBM SPSS, version 20) for quantitative analysis. Descriptive statistics of mean and standard deviation (SD) were applied. The chi-square statistic was used to determine any differences in sleep quality reported by patients and staff. Missing data related to environmental monitoring (noise, light, temperature and humidity) was managed via a data reduction method which deleted cases which involved missing data. To replace missing data an additional night (9 h) of environmental monitoring was undertaken to replace missing data. This method was applied in three cases. The interview responses from the patients and nurses were transcribed from the interview form, and underwent content analysis using NVivo (10 QSR). The coding of the responses was continuous and iterative, with responses coded to emerging themes that were then synthesised into smaller theme matrices. The codes identified were descriptive (rather than interpretative) of the responses in an effort to reduce researcher bias.

## Results

### Demographic profiles

A total of 144 patients (4–10 per clinical unit) (53.5% female) were interviewed across the 15 clinical units, with a mean age of 64.2 years (SD 17, Min-Max, 18 to 90 years,). The mean duration of patient hospital admission at the time of the interview was 15.5 days (SD 34.8, Min-Max, 1 to 275 days,). The Nurses’ Self-report Form was completed by 81 nursing staff (response rate 52%); 88% were registered nurses and 12% were enrolled nurses (nursing staff without tertiary qualifications), which reflected the usual staffing for night duty in this hospital.

### Sleep duration and quality

The patients reported a significant reduction in mean sleep duration (5.3 h, SD 2.33 h, Min-Max, 0 to 10 h,) compared to mean sleep duration at home (7.1 h, SD 1.78 h, Min-Max, 3 to 15 h; *p* < 0.01). A mean decrease in nocturnal sleep duration of 1.8 h was reported by patients during the hospital admission.

**Fig. 1:**
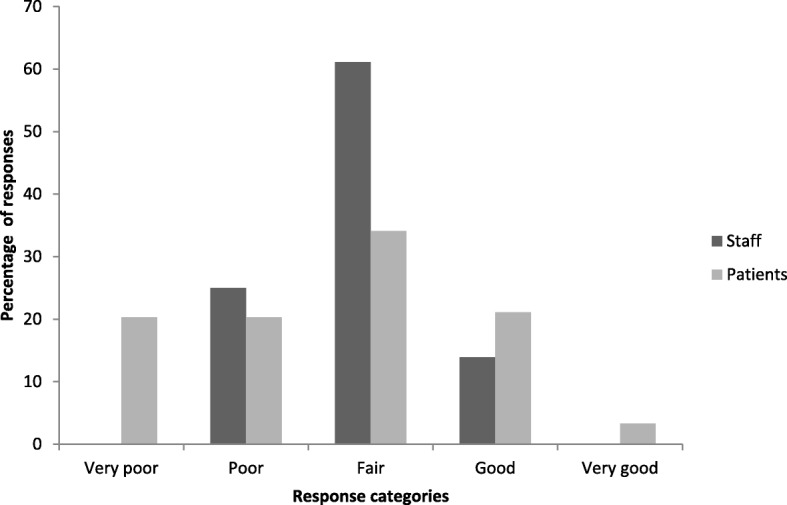
Comparison of Nursing Staff and Patients’ self-reported sleep quality in Hospital. Legend: Nursing staff observational assessment of patient sleep quality did not reflect patients self-reported sleep quality, indicating considerable discordance and as a surrogate assessment may not be reflective of the patients experience

The quality of patient sleep reported by patients and perceived by nursing staff differed significantly (Fig. 1). More patients reported their sleep quality to be poor to very poor (41.6%, *p* < 0.005) compared to nursing staff, with 34.2% of patients compared to 61.1% of nursing staff rating their sleep as fair and 24.2% of patients versus 13.9% of nursing reporting quality sleep as being good. Comparatively, nursing staff were more likely to assess patient sleep as being fair (*p* < 0.3).

### Sleep disturbing factors

The information derived from the patient interviews identified four primary sleep-disturbing factors with clinical care and environmental noise the major sources of sleep disturbance (Fig. 1). Patients reported staff as the primary source of noise, with the main foci for noise being the nursing stations in the clinical environment. Although the interviewees identified the noise made by nursing staff as a contributing factor to sleep disturbance, patients’ responses suggest there was some expectation that their sleep would be negatively affected in hospital. Patients’ comments included: *‘Can’t be helped.’(P10); ‘Not sure if anything can be done.’(P21); ‘Not really, the nurses need to do stuff.’(P95).*

**Fig. 2:**
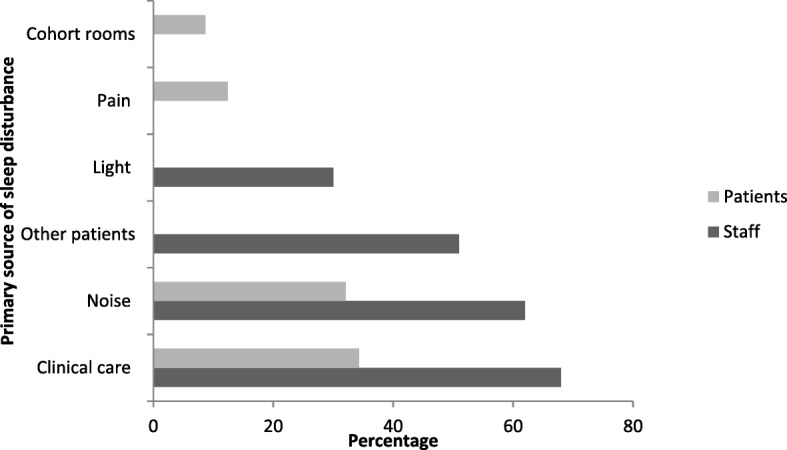
Comparison of Nursing Staff and Patients’ identified factors contributing to sleep disturbance. Legend: The core themes identified by nursing staff and patients as factors that contributed to sleep distance. The combination of clinical care and noise was acknowledge by staff as a disruptive factor and an issue reported by patients. Factors such as pain and cohort rooms were identified by patients as issues that impacted on sleep, but not reported by nursing staff as issues impacting on sleep

While the nursing staff acknowledged that noise in the clinical environment adversely affected patients’ quality of sleep, they perceived that the main source of noise in the clinical environment was ‘other patients’ (Fig. 2)*.* Like the patients, the nursing staff reported that nocturnal noise was an expected and accepted aspect of hospitals. Qualitative responses included: *‘Noise is part of the ward/hospital.’(N16); Cannot be helped, I tell my patients they can rest at home when they are well.’(N19); ‘Unable to make anything quieter (N12); and ‘A lot, but some noises are not practical to minimise in this context’ (N46).*

Some nursing staff respondents acknowledged the impact of noise on patients: *‘Noise is very disturbing for patients, most patients complain’ (N81).* Responses alluded to the impact clinical staff and colleagues behaviours had on the nocturnal environment of wards within the study: *Staff changing from days to night need to remember it is night and make less noise (N18); Try to keep noise down but difficult. I ask wardsmen to try and keep noise down (N 24); Noise can be controlled if everyone co-operates (N54); and Some avoidable with staff attitude change. (N58).*

In addition, nursing staff (65%) reported the exogenous factors of exposure to artificial lighting as the primary cause for patients’ poor quality sleep, compared with 22% reporting offensive odours and 18% reporting a cold clinical environment as the perceived primary sleep disturbing factors for patients.

#### Environmental monitoring

**Fig. 3:**
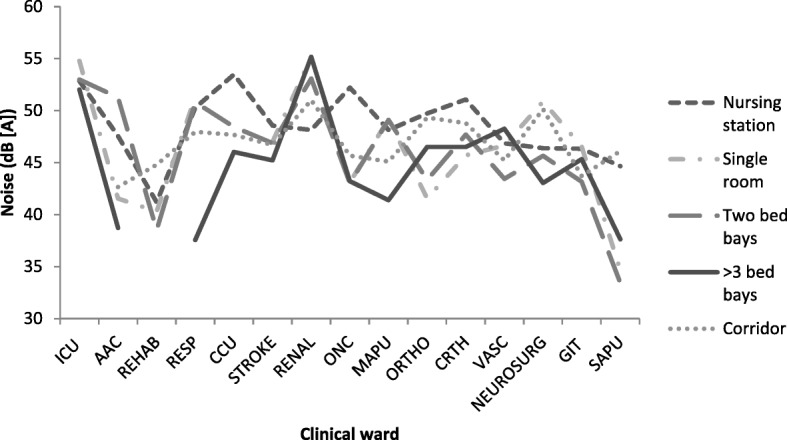
Mean noise levels recorded per monitoring location in the clinical wards. Legend: Noise levels had a similar pattern throughout the clinical areas, with single patient rooms having comparable noise levels to shared patient rooms. Nursing stations within all clinical wards was identified as being the site of considerable noise over the monitoring period

Noise levels were found to be elevated in all clinical areas. The intensive care unit was found to have the highest mean noise level of 53.22 dB(A) (SD 5.72), with the nursing stations in all clinical areas demonstrating the highest percentage (18.7%) of protracted elevations in noise levels greater than 60 dB(A) (Fig. 3). Noise ascensions greater than 70 dB(A) occurred between one and 45 times per hour during the monitoring period. The nursing station and corridors in all clinical wards registered the highest noise levels. Different noise levels were recorded at the four measurement time points with a reduction in noise ranging from 3 to 6 dB between the hours of 01:00 and 05:00, but levels all still exceeding WHO recommendations. Noise was highest in the clinical areas between 22:00 and 24:00 and 05:00 to 07:00 with the highest noise levels recorded in the intensive care unit (98.3 dB[A]), surgical ward (103.3 dB[A]) and medical ward (102 dB[A]).

Observers reported that the four most frequent nocturnal noise sources as staff, with a mean noise occurrence of 34.99 (SD 25.16) times per hour; alarm noise (M 13.81, SD 30.19); doors (M 7.95, SD 8.02) and miscellaneous machine noises (M 6.69, SD 13.43). Noise generated specifically by patients was identified as the fifth most common event, with snoring reported as the major patient-generated noise source (M 5.47, SD 8.97/per hour). The surgical assessment and planning unit (SAPU) had the lowest occupancy rate of all wards at the time of monitoring, which may account for the low noise levels recorded in patient care areas.

### Light

**Fig. 4:**
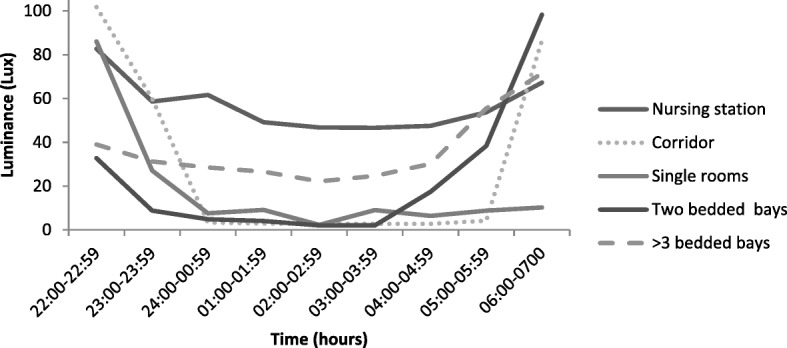
Mean luminance levels recorded per monitoring location. Legend: Exposure to light over the monitoring period displayed a consistent pattern of higher artificial light levels between 22:00 h to 24:00 and again from 05:00 h to 07:00 h. This is consistent with the changeover of staff between the shifts and increased clinical activity

Luminance levels overnight were found to be low (< 100 Lux) throughout the clinical areas and in patient rooms. The highest level of nocturnal illuminance was recorded at the nursing station for all clinical wards, with a minimum luminance of 37.18Lux and a maximum exposure of 150.50 Lux The luminance patterns overnight mirrored the pattern of measured nocturnal noise, with the lowest light levels recorded between 01:00 and 05:00 h, and the highest levels recorded between 22:00 and 24:00 h, and after 05:00 h (Fig. 4).

### Temperature

Recorded temperature varied little overnight across the clinical wards and locations (22.64 to 22.27 °C). Humidity levels remained higher than environmental temperature until the early hours of the morning (02:00 h), before declining by 1.5% and becoming lower than the environmental temperatures. Environmental temperature was reported by 40% of nursing staff as being too cold, although only 18% considered the environmental temperature to be a factor that contributed to patient sleep disturbance. However, only 5% of responding clinical staff reported providing a warm blanket as a sleep promoting strategy. Comparatively, only 4% of patients commented on the environmental temperature as a factor that they perceived disrupted their sleep.

## Discussion

The findings of this study confirm that patients’ sleep duration and quality are compromised during hospital admissions. However, comments provided by patients reveal that they expect to experience sleep disruption in hospital, and view this as an acceptable aspect of healthcare. Given the negative effects of sleep deprivation on patients, this is cause for concern. It could be argued that hospital staff fail in their duty of care as there is increasing evidence that the dangers posed by reduced sleep duration and poorer quality of sleep are as great as those posed by clinical events such as falls, infections and medication errors.

Sleep duration and quality could be considered to be another vital sign worthy of monitoring. In our study, the assessment of sleep quality differed significantly between patients’ self-reports and the perceived quality by nursing staff working in the clinical environment, with staff overestimating patients’ quantity and quality of sleep. Although our finding were not derived from linked assessment, it affirms the findings of other studies where staff reports, using validated instruments such as the Richard-Campbell Sleep Questionnaire (RCSQ), overestimate the quantity and quality of patients’ sleep when compared to patients’ reports and biophysiological measures such as polysomnography [28–31]. Implementing patient self-reporting monitoring of sleep via validated sleep assessment tools such as the RCSQ and motion accelerometer monitoring such as actigraphy, may provide clinicians with greater insight regarding patients’ sleep duration and quality.

The two main factors associated with sleep disturbance in this study were the need to provide clinical care and noise, primarily generated by nursing staff. The latter was consistently reported by patients and nursing staff, and supported by objective environmental monitoring. The ambient noise levels recorded in the clinical environments consistently exceeded the 30 dB(A) WHO recommendation by as much as 82.6%, and were characterised by sporadic noise ascensions in all clinical areas [18]. Previous polysomnographic studies reported that noise levels between 40 and 45 dB(A) are associated with a 10 to 20% probability of awakening, with noise sources over 50 dB(A) producing electroencephalographic changes that indicate arousal [32]. Such noise levels mean that patients are unlikely to achieve the critical sleep stages (slow wave sleep and rapid eye movement) that are important for physiological healing and psychological wellbeing.

Noise in all clinical wards in this study was primarily attributed to staff and in particular to activities at the nursing station. The most disruptive noises reported by patients were directly or indirectly controllable by humans such as conversation levels and television volumes.. This is consistent with previous studies suggesting that over 50% of the noise in the environment was generated by humans [33]. Clinical staff undertaking routine activities appear to be unaware of, or underestimate their effect on patients. This lack of awareness may account for nursing staff overestimating patients’ sleep quality.

The noise patterns generated in this study indicated similarities across all clinical areas, independent of clinical acuity, suggesting that traditional ward designs are a factor in noise generation and dispersion. Centrally located nursing stations frequently act as a hub for clinical activity and socialising and this results in greater dispersion of noise throughout the ward. To reduce noise in the clinical environment, both human behaviour and ward design need to be considered.

The two main sleep disrupting factors of clinical care provision and noise are amendable to behavioural modification [34, 35]. Studies reveal that patients can experience a significant number of clinical interactions overnight with Tamburri et al. reporting a mean of 42.7 nocturnal clinical interactions with patients [36]. The ability and need to cluster clinical activities is imperative in order to facilitate patients ability to sleep, however this may necessitate the reconfiguration of care activities and procedures such as daily radiological and pathological investigations. Ensuring an allocation of time whereby minimal to no clinical interactions and procedures are scheduled has been reported as being beneficial in providing patients with a planned period to rest and sleep [37]. Reassuring patients that sleep is important to recovery and supporting clinical staff in rationalising and the scheduling of clinical activities to permit sleep may contribute beneficially to patients overall well-being. Studies of the effect of behaviour modification strategies have demonstrated reductions in noise levels of 6 to 20% [19]. Reported interventions include staff education about the importance of sleep and the use of noise monitoring equipment to alert staff to increases in noise volume [35]. Kol et al. demonstrated a significant reduction in environmental noise levels in an intensive care environment after the introduction of staff education and a rearrangement of the ward environment (67.6 dB[A] pre versus 56 dB[A] post, *p* < 0.05) [38]. In contrast, Nannapaneni et al. reported that a multipronged noise intervention inclusive of education and noise monitoring had a minimal effect on ambient noise levels, but did result in a reduction in the number of noise ascensions [39]. Although further research into behaviour modification approaches to noise reduction is needed, it appears that behavioural modification alone may not be successful in reducing noise levels [40]. Subsequently, a multifaceted approach—including clinical support and changes to care provision, clinical equipment and ward layout and design may be required to provide a nocturnal environment that supports patient sleep while ensuring safe clinical care. Even simple interventions such as providing patients with masks and ear plugs may enhance patient sleep.

Light and temperature were not identified as major impediments to patient sleep quality in this study. While light exposure has been shown to suppress the regulatory hormone responsible for sleep–wake patterns, the extent to which this may be affected in general clinical environments has not been extensively researched. Current research indicates that low-level light intensity of less than 500 Lux for as few as 20 min is sufficient to suppress melatonin secretion [20, 21, 41]. Therefore its potential physiological effects need to be minimised, by ensuring efforts are undertaken to increase bright light exposure during the day, and limit unwanted light during the night—particularly lights generating blue (white light) wavelengths—through the use of dimmers and alternative lighting strategies [42]. Similarly, it is important to ensure that thermoregulatory efficiency is maintained to limit the deleterious effects of temperature on sleep architecture – in particular slow wave sleep and rapid eye movement sleep. The environmental monitoring of the clinical areas in this study showed that mean environmental temperatures were slightly higher than the optimal temperatures (15 to 20 °C) to support sleep [43]. While, patients did not report this as a sleep impeding factor, clinical staff reported the environmental temperature as cold. This may have been reflective of the physiological effects of fatigue associated with night duty.

### Limitations

While the multiple methods of data collection and inclusion of 15 clinical areas are strengths of this study, the results may not be generalisable to all hospitals or all ward areas. Although unlikely, the factors associated with sleep disturbance may be intrinsic to this hospital and patient cohort experience. Factors such as building construction, changes in the acuity of patients and staffing numbers, and the time of year during which environmental monitoring is undertaken can all affect sleep. As the clinical environments in this study were monitored for only two nights and the environmental data were not continuously recorded the findings may not accurately reflect the diversity in clinical activity that occurs over time. Further, the qualitative information derived from patients and clinical staff were not case matched, but rather provided a personal experience and general perception of the nocturnal clinical environment. The fact that monitoring equipment was positioned to minimise its effects on patients and nurses, rather than to maximise its ability to capture peak noise levels may have affected the results.

It also needs to be acknowledged that while most healthy individuals sleep primarily or exclusively at night, it is important to consider that patients requiring hospitalization will likely require some daytime nap periods. This study looks at sleep only in the night-time period 22:00–07:00 h, without the context of daytime sleep considered.

The researchers made no attempt to deceive clinical staff regarding the nature of the study so the influence of the Hawthorne Effect should be considered. The presence of the observer and environmental monitoring equipment in the clinical environment could have altered behaviour among patients and nursing staff seeking to conform to the presumed research objectives. As a result, the findings reported may be an underestimation of the magnitude of the issues that affect sleep.

## Conclusions

This hospital wide study employed patient, nursing staff and environmental monitoring data to explore the sleep experiences of patients admitted to an Australian hospital and determined that sleep quantity and quality are reduced for the majority of patients. As it is well known that sleep is needed for physiological and psychological health, these findings should be of concern to hospital staff and patients. The fact that patients and nursing staff expect, and accept, poorer quality sleep in hospital demonstrates the lack of knowledge about the many and serious adverse effects of sleep reduction and fragmentation.

The primary factors interrupting patients’ sleep were the need for clinical care and noise. Several strategies to reduce the impact of these sleep limiting factors are available, although evidence for their efficacy is equivocal and more research is needed. Clustering care and tailoring care for patients have both demonstrated some positive effects on sleep quality and quantity. This study also found that noise exceeded WHO recommendations regarding ambient and peak noise with staff, other patients and equipment identified as the main sources of noise. Behaviour modification and critical appraisal of equipment and reconfiguration of the clinical environment may reduce the burden of noise. The perceptions by both nurses’ and patients’ that lost sleep can be recouped when they get home is of particular concern and demonstrates the poverty of understanding about the therapeutic value of sleep indicating that both clinicians and patients require education about the importance of sleep.

The findings of this study provided the impetus for the development of clinical guidelines and education to enhance awareness of sleep physiology among our clinical staff and the evaluation and appraisal of equipment, ward design and clinical practice in order to promote sleep as an important facet to recovery.

## Abbreviations

°C: Celsius
ACT: Australian Capital Territory
dB: Decibels
dB(A): Decibels in the A weighted scale
GB: Gigabyte
Hz: Hertz
IBM: International Business Machines
KHz: Kilohertz
Lux: Illuminance
M: Mean
ms: Milliseconds
N: Nurse (N18 - nurse number 18)
P: Patient (P10 – patient number 10)
QSR: QSR International
RCSQ: Richards-Campbell Sleep Questionnaire
SAPU: Surgical Assessment and Planning Unit
SD: Standard deviation
SDL: Sound detection limit
SPSS: Statistical Packages for Social Sciences
UK: United Kingdom
WHO: World Health Organisation
